# Adrenal suppression in patients taking inhaled glucocorticoids is highly prevalent and management can be guided by morning cortisol

**DOI:** 10.1530/EJE-15-0608

**Published:** 2015-11

**Authors:** Conor P Woods, Nicola Argese, Matthew Chapman, Christopher Boot, Rachel Webster, Vijay Dabhi, Ashley B Grossman, Andrew A Toogood, Wiebke Arlt, Paul M Stewart, Rachel K Crowley, Jeremy W Tomlinson

**Affiliations:** 1 Oxford Centre for Diabetes Endocrinology and Metabolism (OCDEM), NIHR Biomedical Research Centre, Churchill Hospital, University of Oxford, OX3 7LJ, Headington, Oxford, UK; 1 Centre for Endocrinology, Diabetes and Metabolism, University of Birmingham, University Hospitals Birmingham NHS Foundation Trust, Birmingham, B15 2TH, UK; 2 Department of Endocrinology, Faculty of Medicine and Psychology, St Andrea Hospital, Sapienza University of Rome, Rome, Italy; 3 Department of Biochemistry, Queen Elizabeth Hospital, University Hospitals Birmingham NHS Foundation Trust, Birmingham, B15 2TH, UK; 5 Department of Health Informatics, Queen Elizabeth Hospital, University Hospitals Birmingham NHS Foundation Trust, Birmingham, B15 2TH, UK; 6 Department of Endocrinology, University of Leeds, Leeds, UK

## Abstract

**Context:**

Up to 3% of US and UK populations are prescribed glucocorticoids (GC). Suppression of the hypothalamo–pituitary–adrenal axis with the potential risk of adrenal crisis is a recognized complication of therapy. The 250 μg short Synacthen stimulation test (SST) is the most commonly used dynamic assessment to diagnose adrenal insufficiency. There are challenges to the use of the SST in routine clinical practice, including both the staff and time constraints and a significant recent increase in Synacthen cost.

**Methods:**

We performed a retrospective analysis to determine the prevalence of adrenal suppression due to prescribed GCs and the utility of a morning serum cortisol for rapid assessment of adrenal reserve in the routine clinical setting.

**Results:**

In total, 2773 patients underwent 3603 SSTs in a large secondary/tertiary centre between 2008 and 2013 and 17.9% (*n*=496) failed the SST. Of 404 patients taking oral, topical, intranasal or inhaled GC therapy for non-endocrine conditions, 33.2% (*n*=134) had a subnormal SST response. In patients taking inhaled GCs without additional GC therapy, 20.5% (34/166) failed an SST and suppression of adrenal function increased in a dose-dependent fashion. Using receiver operating characteristic curve analysis in patients currently taking inhaled GCs, a basal cortisol ≥348 nmol/l provided 100% specificity for passing the SST; a cortisol value <34 nmol/l had 100% sensitivity for SST failure. Using these cut-offs, 50% (*n*=83) of SSTs performed on patients prescribed inhaled GCs were unnecessary.

**Conclusion:**

Adrenal suppression due to GC treatment, particularly inhaled GCs, is common. A basal serum cortisol concentration has utility in helping determine which patients should undergo dynamic assessment of adrenal function.

## Introduction

Glucocorticoids (GC) have a diverse array of functions affecting every tissue in the body. GCs are essential for life, and both excess and deficiency are associated with increased mortality (1, 2). In patients with an intact hypothalamo–pituitary–adrenal (HPA) axis, endogenous secretion of cortisol from the adrenal cortex is under the control of adrenocorticotropic hormone (ACTH) from the anterior pituitary (3, 4). The dynamic function of the HPA axis is most frequently assessed in clinical practice using the 250 μg short Synacthen (corticotropin) stimulation test (SST). The SST has been validated against the gold standard of insulin-induced hypoglycaemia to provide an accurate reflection of adrenal cortisol reserve (5), therefore representing the most widely used tool for diagnosing adrenal insufficiency. There are significant challenges to the use of the SST in clinical practice. These challenges include both the staff and time needed to perform the SST, a well-documented recent (2014) shortage of Synacthen and a subsequent significant increase in Synacthen cost.

Endocrine diseases affecting the adrenal or pituitary gland are important causes of primary and secondary causes of adrenal insufficiency respectively (6). However, prescribed GCs can also cause long-term suppression of the HPA axis and, consequently, adrenal atrophy and an inability to mount an adequate cortisol response to stress, rendering patients at an increased risk of adrenal crisis. Adrenal crisis is a medical emergency necessitating hospital admission, resuscitation with i.v. fluids and parenteral GC treatment, and is associated with increased mortality and a poor outcome (7, 8).

The therapeutic indications and clinical benefit associated with GC treatment prescribed for underlying inflammatory conditions are not in doubt. Estimates suggest that up to 3% of the population of the UK and USA are currently taking GC therapy (9, 10). Long-term prescription rates for oral GCs have increased, as has the use of inhaled GC therapy (11). In the majority of cases, the doses of prescribed GCs are sufficient to cause HPA axis suppression (total daily doses >5 mg prednisolone or equivalent) (10). GC prescriptions are often extended for a sustained period of time with a median duration in excess of 4 years; in 25% of the cases, treatment extends beyond 5 years (9). As a result, suppression of the HPA axis may be frequent and potentially overlooked. Dynamic assessment of the HPA axis using the SST has not been widely reported in the literature in the setting of iatrogenic adrenal suppression. Furthermore, the decision to undertake dynamic HPA axis testing in patients taking prescribed GCs is often based upon the presence of symptoms that may be suggestive of adrenal insufficiency, but these can be vague and often non-specific (12). Importantly, there are currently no published data to help guide the clinician to decide in which patients to undertake assessment of adrenal cortisol reserve. We can assume that people taking a regular dose of 5 mg of prednisolone (or equivalent) have compromised adrenal reserve; however, the degree of HPA axis suppression in persons taking inhaled, topical or intra-articular GCs is unknown.

We have performed a detailed retrospective analysis over a 5-year period in a secondary/tertiary care centre. Firstly, we have aimed to determine the prevalence of adrenal insufficiency in patients undergoing SSTs across all medical specialties, and specifically the prevalence of adrenal insufficiency due to prescribed GCs. Secondly, we have analysed whether a morning serum cortisol can guide the clinician as to which patients are likely to have an intact HPA axis, and thus to rationalize further investigation and management.

## Methods

We retrospectively analysed the results of SSTs performed in 2773 patients from the electronic medical record system at the Queen Elizabeth Hospital, University Hospitals Birmingham NHS Foundation Trust, UK. All SSTs were performed in the morning (0900–1200 h), between 1/1/2008 and 31/12/2012 across all specialties in a single large secondary/tertiary care centre in the UK. An i.m. administration of synthetic ACTH [1–24] was used (Synacthen 250 μg, Alliance Pharmaceuticals, Chippenham, UK). In patients treated with oral GCs, these were suspended for 24-h prior to testing. For patients with acute pituitary insult (surgery or apoplexy), the SST was delayed for a minimum of at least 4 weeks after surgery or presentation, as earlier assessment can result in false positive passes, since adrenal atrophy only develops 4 weeks after the insult (13). Clinical characteristics of individuals were recorded, including age, sex, indication for the SST and the use of GC medication (including dose and route of administration). Patients on GC replacement therapy as a consequence of adrenal or pituitary disease were classified according to their underlying endocrine condition rather than in groups associated with GC administration. Similarly, patients with CNS disease (including tumours) were classified separately from those prescribed GC therapy due to the potential for the underlying disease process to impact upon endogenous HPA axis function. Serum cortisol was analysed on a standard automated competitive immunoassay platform (Roche Modular system, Roche) where there is no evidence for cross-reactivity with commonly prescribed inhaled GCs (14). The inter-assay imprecision was <8% for levels between 76 and 925 nmol/l. The basal and stimulated 30-min cortisol samples were analysed in a single batch. Using data from a healthy control population (15) and the same cortisol assay, a 30-min cortisol of ≥550 nmol/l was regarded as demonstrating a satisfactory adrenal reserve and designated a ‘pass’, whereas a 30-min value of <550 nmol/l was designated a ‘fail’.

### Statistical analysis

Results are expressed as means±s.d. for parametric data and medians and inter-quartile ranges for non-parametric data. For comparison of single variables, *t*-tests have been used (or non-parametric equivalents) with paired analysis where appropriate. For analyses involving multiple comparisons, one-way ANOVA with Bonferroni's multiple comparison *post-hoc* correction or, for non-parametric data, Kruskal–Wallis with Dunn's multiple correction tests were used to determine statistical significance. Two-tailed significance was set at *P*<0.05. For comparison of the prevalence of SST failure/pass, *χ* ^2^ analysis was used. To analyse basal cortisol as a predictor of passing the SST, receiver operator characteristic (ROC) curves were generated with true positive results (sensitivity) plotted against false positive (1-specificity). Area under the curve (AUC) of a ROC curve indicates the ability to discriminate a true result, with values of 0.5 showing no discrimination and values of 1.0 equal to perfect discrimination. The ‘best fit’ value of the curve was determined using the Youden index (threshold value for which (Sensitivity+Specificity−100) is maximized). We performed sub-group analysis in the following categories: age, sex (including pre and post-menopause), pituitary disease and inhaled GCs. To determine if the inclusion of repeat tests would influence the discriminatory value of a morning cortisol to predict adrenal reserve, a further analysis including the entire cohort of all 3603 SSTs was performed (see Supplementary Table 1, see section on supplementary data given at the end of this article). Statistical analysis was performed using the GraphPad Prism 6.0 software package (GraphPad Software, Inc. La Jolla, CA, USA).

## Results

Overall, 496 patients (17.9%) failed the SST (Table 1). In cases where the SST had been performed without a confirmed endocrine diagnosis, or in patients not receiving GC therapy, failure rates were low (0–8.8%).

### Adrenal suppression due to prescribed GCs

In total, 404 SSTs were performed to determine adrenal reserve in patients who had been prescribed GC therapy (indications 1 and 2 of Table 1). The prevalence of SST failure in this cohort was 33.2% (134/404). Patients with pituitary, adrenal and CNS disease (indications 3–8 from Table 1) were then excluded. Patients were further subdivided based upon their GC therapy status: those currently taking GCs and those previously exposed to GC therapy (Table 2). Data were compared against individuals who were GC naïve and without established endocrine or CNS disease (*n*=1287). In GC-naïve patients, 7.5% (96/1287) failed the SST. A similar SST failure rate was observed for those patients previously exposed to GC therapy (10.9% (7/64)). However, failure rates were significantly higher in patients currently taking GC therapy (37.4% (127/340) *χ* ^2^ analysis, *P*<0.05). In patients currently taking prescribed GCs, failure rates were highest in those patients taking oral therapy (58.4% (73/125), *P*<0.01).

### HPA axis suppression and inhaled GCs

**Figure 1:**
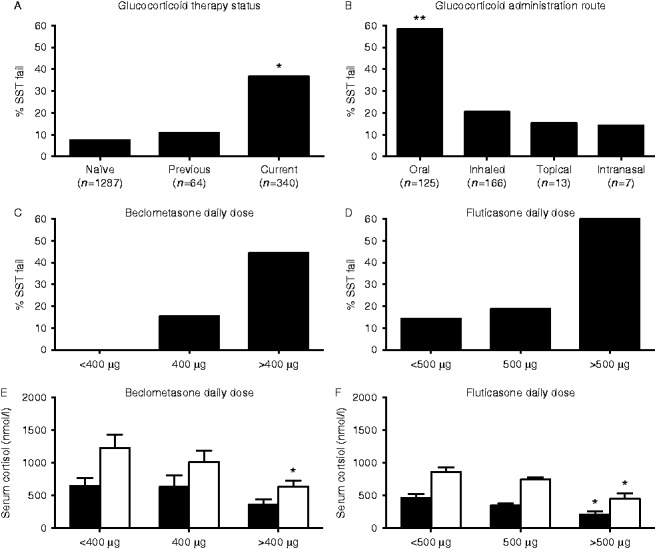
Excluding patients with underlying pituitary, adrenal or CNS disease, current glucocorticoid therapy is associated with increased rates of SST failure (A). SST failure is common across all routes of glucocorticoid administration, although it is most frequent in patients on oral therapy (B). (*P<0.05, **P<0.01). The impact of inhaled glucocorticoid therapy on the prevalence of adrenal suppression (C, D, E and F). Beclometasone and fluticasone administration cause a dose-dependent increase in SST failure rates (C and D) and absolute reductions in basal and 30-min cortisol levels after synthetic ACTH_1__–__24_ stimulation (E and F) (basal serum cortisol=black bars, 30-min cortisol=white bars) (*P<0.05 vs lowest daily dose).

SST failure remained high in patients currently prescribed inhaled GC or topical GC therapy compared to GC-naïve patients (Fig. 1). In patients taking inhaled GCs without additional GC therapy, 34 of 166 patients failed the SST (20.5%). SST failure rates were 21.2% (22/104) for patients taking fluticasone, 16.7% (6/36) for beclometasone, 19.1% (4/21) for budesonide and 2/5 on ciclesonide. Where doses were recorded (beclometasone=28, or fluticasone *n*=79), the prevalence of SST failure was highest in those patients taking the largest doses (Fig. 1C and D). The 30-min serum cortisol levels were significantly lower in patients on the highest doses of both beclometasone and fluticasone in comparison with those patients on lower doses (Fig. 1E and F). Baseline cortisol levels were also suppressed on the highest doses of fluticasone (461±60 vs 205±49 nmol/l, *P*<0.05) (Fig. 1F).

Comparing the SST response between patients taking beclometasone or fluticasone, 30-min cortisol levels were not significantly different at the highest doses of inhaled GCs (445±85 vs 631±98 nmol/l, fluticasone >500 μg/day vs beclometasone >400 μg/day, *P*=NS). However, at lower doses, fluticasone therapy was associated with a greater impairment of 30-min cortisol response (858±70 vs 1229±201 nmol/l, fluticasone <500 μg/day vs beclometasone <400 μg/day, *P*<0.05; 740±37 vs 1009±177 nmol/l, fluticasone 500 μg/day vs beclometasone 400 μg/day, *P*<0.05). There were no significant differences in clinical characteristics in the patient cohorts when comparing across prescribed dose of inhaled GCs between different inhaled GCs or those who failed or passed the SST (data not shown).

### Baseline morning cortisol as a predictor of SST outcome

**Figure 2:**
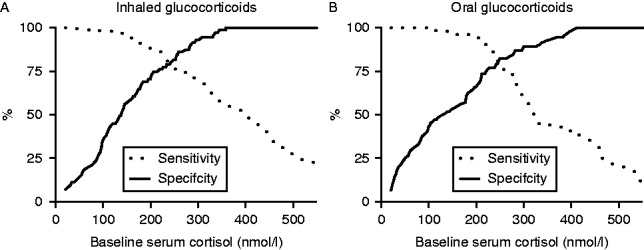
Baseline serum cortisol as a predictor of 30-min cortisol levels during an SST. Baseline serum cortisol is graphed against the % likelihood of passing (specificity=continuous line) or failing (sensitivity=dashed line) the SST. (A) Inhaled glucocorticoids. (B) Oral glucocorticoids.

Baseline serum cortisol in the cohort of 3606 SSTs (2773 patients) correlated significantly with the levels 30 min after ACTH stimulation (Sperman's *ρ*=+0.74 *P*<0.0001). ROC curve analysis of baseline morning cortisol as a predictor for passing the SST showed an AUC of 0.91. Serum cortisol concentration of 506 nmol/l gave 100% specificity for predicting passing the SST and a serum cortisol concentration of 107 nmol/l was 99% sensitive for predicting failure (Table 3). In the 404 patients exposed to GC therapy, a serum cortisol concentration of 410 nmol/l gave 100% specificity for predicting passing the SST and a serum cortisol concentration of 34 nmol/l was 100% sensitive for predicting failure. Also, 166 SSTs were performed on patients taking inhaled GCs. All those with a baseline morning cortisol of 348 nmol/l or higher passed the SST and all those with a baseline serum cortisol concentration of 34 nmol/l or lower failed the SST (Table 3 and Fig. 2A). Adopting these 100% cut-offs (348 and 34 respectively, 83 SSTs (representing 50% of all SSTs undertaken in patients on inhaled GCs) need not have been performed, as the results of the SST could have been predicted form the morning cortisol result alone (Fig. 2A).

The AUC, best-fit cortisol value and cut-off cortisol values for respective specificities and sensitivities according to age, sex, menopausal status and other indications (including endocrine diagnoses) are presented in Supplementary Table 2, see section on supplementary data given at the end of this article.

## Discussion

In this study we have demonstrated a high prevalence of adrenal insufficiency associated with GC treatment in the largest cohort described to date, with an alarming prevalence in those patients taking GC therapy for non-endocrine disease. Indeed, SST failure rates were higher in those taking GC therapy than in those with underlying pituitary disease. GCs are currently prescribed to up to 3% of the population (9, 10, 11) and there is little doubt that GC excess is associated with significant adverse effects and suppression of the HPA axis (16). Furthermore, epidemiological data from general practice records has demonstrated a link between GC prescriptions and the risk of cardiovascular events and heart failure (17).

Adrenal suppression is considered unusual where GCs have been prescribed for <2 weeks (18) but is more likely following prolonged administration (19). However, a recent prospective study in patients prescribed high-dose oral prednisone (40 mg) identified evidence of adrenal suppression in >60% of patients after 6 days of therapy (20). Some studies have suggested that adrenal suppression is not tightly linked to the duration and dose of oral GCs (16, 21); however, this is not the case in all studies (22) and this may well reflect inter-individual variability in GC action and metabolism, including the activity of CYP3A4, the major pathway for the inactivation of most prescribed GCs (23, 24, 25, 26).

Within our cohort, more than 20% of patients taking prescribed inhaled GCs failed the SST, which is consistent with some but not all of the published literature (27, 28, 29). The variability in results may reflect the use of inappropriate assessments of HPA axis and paucity of dynamic testing including SSTs; isolated measurements of serum cortisol are difficult to interpret and can be misleading. We and others have previously documented the prevalence of adrenal insufficiency in relatively small cohorts of patients (*n*=33) taking inhaled GCs for respiratory disease and demonstrated SST failure rates of up to 48% (30).

Our data have shown a dose dependency in the SST response with inhaled GCs and are in agreement with evidence from the published literature suggesting that adrenal suppression is most frequent at the highest doses of inhaled GCs (31), although we did observe suppression in some patients taking lower doses. In addition, it is important to recognize that adrenal suppression can persist for prolonged periods after discontinuation of GC therapy (32). Whilst we have been able to demonstrate the magnitude of a clinical problem that is more extensive than is widely appreciated, it is fundamentally important to understand its clinical consequences. Current clinical data in this regard are lacking. We have previously shown that decreased adrenal reserve as a consequence of inhaled GCs is associated with reduced quality of life in patients with bronchiectasis (30) and the use of inhaled GCs in patients with chronic obstructive pulmonary disease (COPD) is known to increase the risk of type 2 diabetes and pneumonia (11, 33). In the setting of intensive care, a significant proportion of patients have functional adrenal insufficiency and this has been associated with a poorer outcome (34). Taken together, these observations raise the intriguing possibility that poor clinical outcome in patients on inhaled GCs may be due at least in part to HPA axis suppression, adrenal insufficiency and the consequent inability to mount an adequate cortisol stress response, and this has significant implications for the therapeutic approach to these patients (Supplementary Table 3 and Supplementary Figure 1, see section on supplementary data).

**Figure 3:**
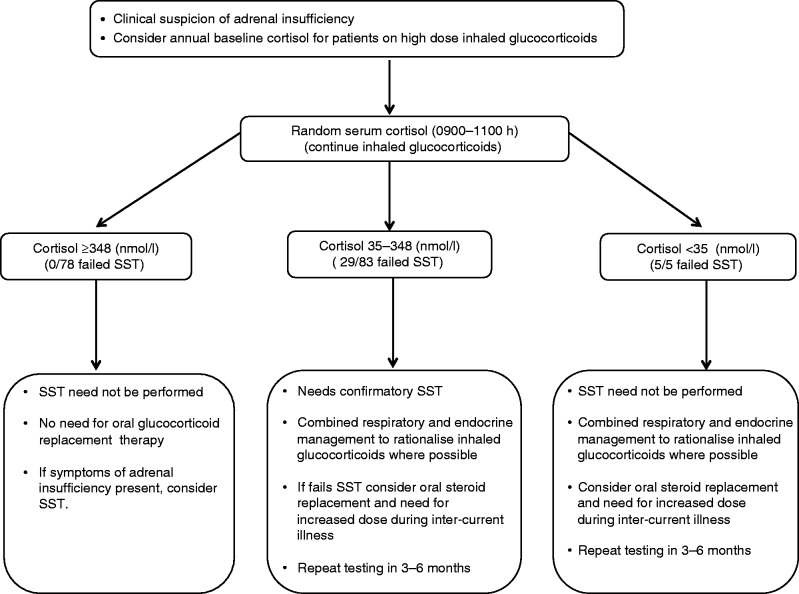
Putative algorithm to aid in the rationalization of assessment of the HPA axis in patients taking inhaled glucocorticoids.

Routine screening for adrenal insufficiency in the context of inhaled GCs is not currently recommended. The magnitude of the clinical problem may be daunting, and making a case for routine assessment of HPA axis function in all patients on inhaled GCs may be prohibitive considering the workload and cost implications. To date, dynamic stimulation tests such as the SST and the ITT are considered mandatory for reliable investigation of adrenal reserve (13, 35, 36). The utility of a basal cortisol in predicting adrenal reserve has been examined previously (37, 38). Using a single morning cortisol as the first screening step has the potential, in some circumstances, to reduce the need for dynamic tests and as a consequence to decrease cost and workload and reduce unnecessary tests for patients. In addition, the recent well-publicized worldwide shortage of Synacthen has highlighted the role that alternative assessments of HPA axis function may play. A baseline cortisol result below 100 nmol/l is considered highly indicative of adrenal insufficiency (37, 39), although this is not universally accepted (35, 40). Different basal cortisol levels, ranging from 285 to 500 nmol/l, have been proposed as predictive of adequate adrenal reserve (35, 37, 39, 40, 41, 42, 43). However, these studies have been small (*n*=197, 761, 68, 679, 210, 54 and 166 respectively) and in many cases have not been able to systematically examine variable cortisol level cut-offs or determine the impact of underlying aetiology and specifically the clinical problem of iatrogenic adrenal insufficiency. Through our systematic approach to assessing adrenal function in a large cohort of patients, we are now able to propose an algorithm (Fig. 3) that is likely to have significant clinical utility and is safe. In patients prescribed inhaled GCs and in the absence of symptoms of adrenal insufficiency, we recommend measuring annual baseline serum cortisol concentrations as a useful tool in assessing adrenal GC reserve. A baseline serum cortisol >348 nmol/l is suggestive of an intact adrenal response to stress. A baseline cortisol of <35 nmol/l suggests the need for physiological oral hydrocortisone replacement and, if possible, a reduction in inhaled GC doses. Baseline serum cortisol concentrations between 35 and 348 nmol/l suggest the need for further investigation, including an SST and possible oral GC replacement. We deliberately identified a cut-off with 100% specificity such that we can be confident not to miss unrecognized adrenal insufficiency. Adopting this algorithm would mean that 50% of the SSTs on patients taking inhaled GCs need not have been performed. A similar approach could be used in patients with underlying adrenal or pituitary pathology, limiting unnecessary test and the potential for significant cost savings.

The management of patients with adrenal insufficiency in the context of endocrine disease has been reviewed extensively (6). However, there are currently no guidelines or published studies that have determined the optimal management strategy for those patients with adrenal insufficiency due to prescribed GCs. For those on oral therapy, continuation of treatment as clinically indicated is appropriate, followed by weaning of the dose if the duration has been longer than 2 weeks. Once the daily doses have been reduced to those approaching physiological levels (equivalent of prednisolone 5 mg/day), and assuming that GC therapy is no longer indicated for their initial prescribed purpose, an SST should be performed and, if passed, the GCs can be safely stopped. If, however, the SST is failed, we would normally initiate hydrocortisone replacement therapy with appropriate ‘sick day rules’ with regards to increasing dose at times of stress or inter-current illness, provide the patient with information and a ‘steroid card’ and perform repeat testing after 3–6 months.

For those patients taking inhaled GCs, the situation is more complex, as the inhaled GC cannot provide the adequate systemic actions that are needed at times of stress. A combined respiratory and endocrinology approach is appropriate. If possible, inhaled GC doses may be reduced, but this will be entirely dependent upon the underlying respiratory condition. For those patients who fail the SST, we would advocate daily hydrocortisone replacement (including advice with regards to ‘sick day rules’ and provision of an emergency ‘steroid card’ as described above). Repeat SST should be performed, especially if modification to the inhaled GC therapy has been undertaken to assess the potential return of HPA axis function.

There are limitations with the current analysis, although it does represent a cross-sectional analysis of unselected clinical data across all medical specialties. The data are retrospective and the decision to perform the SST was based on clinical indication as assessed by the individual clinician leading to a potential positive selection bias that may overestimate prevalence rates. Duration of GC treatment is likely to be important, as is the length of time since cessation of GC therapy. However, data from our electronic patient records does not allow accurate assessments of the duration of therapy or the time since therapy had been discontinued. The cut-off levels in this paper relate specifically to the assay used, and interpretation of these data should be made in the context of local cortisol assays as well as the methodology for the SST (i.m. vs i.v. Synacthen and 30 vs 60 min cortisol values). We advocate utilising the i.m. high dose Synacthen test, as it performs well in clinical practise (13, 44) and has been validated against the gold standard (insulin-induced hypoglycaemia) (5). In addition, robust longitudinal clinical outcome data was not available from our electronic database, which is of fundamental importance.

The SST failure rate in GC-naïve patients without established endocrine or CNS disease was higher than might have been expected (7.5%). The explanation for this is not clear and may relate to new diagnoses of endocrine (or other, e.g. malignancy) disease that had not been fully investigated prior to the SST being requested, although the aetiology in many remains unexplained. Importantly, some of these tests were performed in the intensive care setting where the underpinning mechanisms causing compromised adrenal reserve are still unknown.

In summary, the prevalence of adrenal insufficiency due to prescribed GCs is high and almost certainly represents the commonest cause of compromised adrenal function. Importantly, we have highlighted the potent, dose-dependent ability of inhaled GCs to suppress endogenous HPA axis function, and identified the utility of a morning cortisol level in guiding the clinician as to which patients may need dynamic assessment of adrenal reserve. Futures prospective studies are needed to accurately define the clinical consequences of adrenal suppression for patients prescribed inhaled GC therapy and determine the optimal medical management for these patients.

## Supplementary data

This is linked to the online version of the paper at http://dx.doi.org/10.1530/EJE-15-0608.

## Author contribution statement

C P Woods, N Argese and M Chapman analysed the data and wrote the manuscript. C Boot and R Webster collected the biochemical data and helped to write the manuscript. V Dabhi collected the data from the electronic patient records and analysed the data. A A Toogood, A B Grossman, W Arlt, P M Stewart and R K Crowley scrutinized, analysed and interpreted the data and helped to write the manuscript. J W Tomlinson designed the study, analysed and interpreted the data and wrote the manuscript.

## Figures and Tables

**Table 1:** The results of SSTs in 2773 patients divided according to indication.

	**Description of indication**	**Total** (*n*)	**% Pass** (*n*)	**% Fail** (*n*)
1	Treatment with inhaled, intra-nasal or topical glucocorticoids	228	75.4 (172)	24.6 (56)
2	Treatment with oral or i.v. glucocorticoids	176	55.7 (98)	44.3 (78)
3	Post-operative assessment after pituitary surgery (without radiotherapy)	195	70.3 (137)	29.7 (58)
4	Post-operative assessment after pituitary surgery (with radiotherapy)	59	71.2 (42)	28.8 (17)
5	Pituitary adenoma (without surgery or radiotherapy)	264	89.8 (237)	10.2 (27)
6	Other conditions affecting the pituitary	175	77.1 (135)	22.9 (40)
7	Other tumours of the CNS	315	74.9 (236)	25.1 (79)
8	Adrenal disease (CAH, Addison's disease, adenoma, carcinoma)	74	39.2 (29)	60.8 (45)
9	Co-existent autoimmune disease (thyroid disease, type 1 diabetes mellitus, premature ovarian failure, vitiligo)	113	92.0 (104)	8.0 (9)
10	Hyponatraemia or hyperkalaemia	68	91.2 (62)	8.8 (6)
11	Hypoglycaemia	32	100 (32)	0 (0)
12	Hypotension, syncope, collapse	173	96.0 (166)	4.0 (7)
13	Fatigue, weight loss, malaise	178	92.7 (165)	7.3 (13)
14	Other indications, including critical care admission or not specified	723	91.6 (662)	8.4 (61)
		2773	82.1% (2277)	17.9% (496)

**Table 2:** The impact of glucocorticoid therapy status upon SST results. Indications are as follows: (1) treatment with inhaled, nasal or topical glucocorticoids; (2) treatment with i.v. or oral glucocorticoids; (3) post-operative assessment after pituitary surgery (without radiotherapy); (4) post-operative assessment after pituitary surgery (with radiotherapy); (5) pituitary adenoma (without surgery or radiotherapy); (6) other conditions affecting the pituitary; (7) other tumours of the CNS; (8) Adrenal disease (CAH, Addison's disease, adenoma, carcinoma); (9) co-existent autoimmune disease (thyroid disease, type 1 diabetes mellitus, premature ovarian failure, vitiligo); (10) hyponatremia or hyperkalaemia; (11) hypoglycaemia; (12) hypotension, syncope, collapse; (13) fatigue, weight loss, malaise; and (14) other indications, including critical care admission or not specified.

**Indication**	**Glucocorticoid naive**			**Previous glucocorticoid therapy**			**Current glucocorticoid therapy**		
*n*	% Pass (*n*)	% Fail (*n*)	*n*	% Pass (*n*)	% Fail (*n*)	*n*	% Pass (*n*)	% Fail (*n*)
1	0	0	0	13	84.6 (11)	15.4 (2)	215	74.9 (161)	25.1 (54)
2	0	0	0	51	90.2 (46)	9.8 (5)	125	41.6 (52)	58.4 (73)
3	103	89.3 (92)	10.7 (11)	9	100 (9)	0 (0)	83	43.4 (36)	56.6 (47)
4	44	86.4 (38)	13.6 (6)	2	100 (2)	0 (0)	13	15.4 (2)	84.6 (11)
5	238	95.0 (226)	5.0 (12)	3	33.3 (1)	66.7 (2)	23	43.5 (10)	56.5 (13)
6	132	93.2 (123)	6.8 (9)	1	100 (1)	0 (0)	42	26.2 (11)	73.8 (31)
7	222	95.0 (211)	5.0 (11)	18	83.3 (15)	16.7 (3)	75	13.3 (10)	86.7 (65)
8	34	76.5 (26)	23.5 (8)	0	0	0	40	7.5 (3)	92.5 (37)
9	113	92.0 (104)	8.0 (9)	0	0	0	0	0	0
10	68	91.2 (62)	8.8 (6)	0	0	0	0	0	0
11	32	100 (32)	0 (0)	0	0	0	0	0	0
12	173	96.0 (166)	4.0 (7)	0	0	0	0	0	0
13	178	92.7 (165)	7.3 (13)	0	0	0	0	0	0
14	723	91.6 (662)	8.4 (61)	0	0	0	0	0	0

**Table 3:** SST results with AUC, best-fit serum cortisol (max (Sensitivity+Specificity−100)) and sensitivities and specificities for serum cortisol concentrations (nmol/l).

**Group**	**AUC**	**Best-fit cortisol** (nmol/l)	**Specificity** (%)			**Sensitivity** (%)		
95	99	100	95	99	100
All patients (*n*=2773)	0.91 (0.89–0.92)	221 (86%sens+77%spec)	347	461	506	164	107	<20
All current GC use (*n*=404)	0.92 (0.89–0.94)	220 (78%sens+88%spec)	331	386	410	168	63	34
Inhaled GCs (*n*=166)	0.90 (0.85–0.95)	308 (70%sens+94%spec)	342	347	348	146	62	34
Oral GCs (*n*=143)	0.89 (0.84–0.94)	213 (74%sens+94%spec)	383	405	413	198	115	114
